# Cognitive screening instruments for dementia: comparing metrics of test limitation

**DOI:** 10.1590/1980-57642021dn15-040005

**Published:** 2021

**Authors:** Andrew J. Larner

**Affiliations:** 1 Cognitive Function Clinic, Walton Centre for Neurology and Neurosurgery – Liverpool, United Kingdom.

**Keywords:** cognitive screening, dementia, diagnosis, limitations, memory clinic, rastreio cognitivo, demência, diagnóstico, limitações, clínica de memória

## Abstract

Cognitive screening instruments (CSIs) for dementia and mild cognitive impairment are usually characterized in terms of measures of discrimination such as sensitivity, specificity, and likelihood ratios, but these CSIs also have limitations.

**Objective:**

The aim of this study was to calculate various measures of test limitation for commonly used CSIs, namely, misclassification rate (MR), net harm/net benefit ratio (H/B), and the likelihood to be diagnosed or misdiagnosed (LDM).

**Methods:**

Data from several previously reported pragmatic test accuracy studies of CSIs (Mini-Mental State Examination, the Montreal Cognitive Assessment, Mini-Addenbrooke’s Cognitive Examination, Six-item Cognitive Impairment Test, informant Ascertain Dementia 8, Test Your Memory test, and Free-Cog) undertaken in a single clinic were reanalyzed to calculate and compare MR, H/B, and the LDM for each test.

**Results:**

Some CSIs with very high sensitivity but low specificity for dementia fared poorly on measures of limitation, with high MRs, low H/B, and low LDM; some had likelihoods favoring misdiagnosis over diagnosis. Tests with a better balance of sensitivity and specificity fared better on measures of limitation.

**Conclusions:**

When deciding which CSI to administer, measures of test limitation as well as measures of test discrimination should be considered. Identification of CSIs with high MR, low H/B, and low LDM, may have implications for their use in clinical practice.

## RESUMO

Os instrumentos de rastreio cognitivo (IRCs) para demência e comprometimento cognitivo leve são geralmente caracterizados em termos de medidas de discriminação, como sensibilidade, especificidade e razões de probabilidade, mas esses IRCs também têm limitações.

**Objetivo:**

Calcular várias medidas de limitação de testes para IRC comumente usados, a saber: taxa de classificação incorreta; relação entre dano líquido e benefício líquido; e probabilidade de diagnóstico ou diagnóstico incorreto.

**Métodos:**

Os dados de vários estudos de precisão de teste pragmático de IRC relatados anteriormente (MMSE, MoCA, MACE, 6CIT, AD8, TYM, Free-Cog) e realizados em uma única clínica foram reanalisados para calcular e comparar a taxa de classificação incorreta, o dano líquido para a relação de benefício líquido e a probabilidade de diagnóstico ou diagnóstico incorreto para cada teste.

**Resultados:**

Alguns IRC com sensibilidade muito alta, mas baixa especificidade para demência, tiveram desempenho ruim em medidas de limitação, com altas taxas de classificação incorreta, baixo prejuízo líquido para relações de benefício líquido e baixa probabilidade de diagnóstico ou diagnóstico incorreto; alguns tinham probabilidades de favorecer o diagnóstico incorreto ao invés do diagnóstico. Testes com melhor equilíbrio de sensibilidade e especificidade saíram-se melhor nas medidas de limitação.

**Conclusões:**

Ao decidir qual IRC administrar, as medidas de limitação, bem como as medidas de discriminação do teste, devem ser consideradas. A identificação de IRC com alta taxa de classificação incorreta, baixa relação de prejuízo e benefício e baixa probabilidade de diagnóstico ou diagnóstico incorreto pode ter implicações para seu uso na prática clínica.

## INTRODUCTION

Like all screening and diagnostic tests, cognitive screening instruments (CSIs) are usually characterized in terms of the conditional probabilities of sensitivity (Sens) and specificity (Spec), where Sens (or true positive rate, TPR) is the correct identification of those with dementia or cognitive impairment and Spec (or true negative rate, TNR) is the correct exclusion of those without disease (see Table 1 for definitions of metrics discussed in this study, their formulae, and score ranges). Information from both Sens and Spec may be combined in metrics such as the Youden index (Y) and positive and negative likelihood ratios (LR+, LR−), of which the latter may be qualitatively classified as causing slight, moderate, large, or very large change in probability of disease or its absence.^1^ Sens and Spec are suggested key words for reports of diagnostic test accuracy studies in dementia (STARDdem)^2^ and LRs were used as the basis for recommendations made by the UK National Institute for Health and Care Excellence for tests suitable for dementia.^3^ Systematic reviews and meta-analyses of CSIs, for example, those produced by the Cochrane Dementia and Cognitive Improvement Group,^4^ typically quote summary test Sens, Spec, and LRs.

**Table 1.** Metrics, formulae, and ranges for measures of test discrimination and limitation.

Metric	Formula	Range
Measures of test discrimination		
Sens, TPR	TP/(TP+FN)	0–1, higher better
Spec, TNR	TN/(TN+FP)	0–1, higher better
Y	Sens+Spec−1	0–1, higher better*
LR+	Sens/(1−Spec)=TPR/FPR	1–∞, higher better
LR−	(1−Sens)/Spec=FNR/TNR	0–1, lower better
NND	1/Y	1–∞, lower better
NNM	1/Inacc	1–∞, higher better
Measures of test limitation		
FNR	FN/(TP+FN)=1−Sens	0–1, lower better
FPR	FP/(TN+FP)=1−Spec	0–1, lower better
Inacc	(FP+FN)/(TP+FP+FN+TN)	0–1, lower better
EOR	TP×FP/FN×TN	0–∞, lower better
MR	FNR+FPR=2−(Sens+Spec)	0–2, lower better
net H/B ratio	pre-test odds×LR+	Higher better if FN deemed worse than FP
LDM	NNM/NND=Y/Inacc	−1–∞, <1 favors misdiagnosis, >1 favors diagnosis

Sens, TPR: Sensitivity; Spec, TNR: Specificity; Y: Youden index; LR+: positive likelihood ratio; LR−: negative likelihood ratio; NND: number needed to diagnose; NNM: number needed to misdiagnose; FNR: false negative rate; FPR: false positive rate; Inacc: Inaccuracy; EOR: error odds ratio; MR: misclassification rate; net H/B ratio: net harm/net benefit ratio; LDM: likelihood to be diagnosed or misdiagnosed. *Values of Y may in fact range from −1 to +1, as is evident from the formula. However, most values of Y would be expected to fall within the range 0 (worthless test, no diagnostic value) to 1 (perfect diagnostic value, no false positives or false negatives), with negative values occurring only if the test were misleading, that is, the test result was negatively associated with the true diagnosis.

Like all screening and diagnostic tests, CSIs are not perfect. They have shortcomings, inadequacies, or failures, which may be termed “limitations.” Tests have potential harms (misdiagnosis) as well as benefits (correct diagnosis). The limitations comprise failure to identify dementia or cognitive impairment when it is in fact present and identifying these states when they are in fact absent. These rates, respectively, false negative (FNR) and false positive (FPR), are implicit in the measures of Sens and Spec since, by the principle of summation, they are their complements or negations (FNR=1−Sens; FPR=1−Spec). Other metrics of test limitation include inaccuracy (Inacc; also sometimes known as fraction incorrect or error rate) and error odds ratio, although these measures are seldom used in clinical practice.

Other metrics of test limitation, which, like all those already mentioned, may be derived from the 2×2 contingency table of diagnostic test accuracy studies, form the subject of the current study. These are the misclassification rate (MR), the net harm/net benefit ratio (H/B), and the likelihood to be diagnosed or misdiagnosed (LDM).

The sum of FNR and FPR is used here to define the MR, following the usage of Perkins and Schisterman.^5^ (Confusingly, this term has also been sometimes used interchangeably with Inacc.) Minimization of MR is used in some of the methods for setting a test threshold from inspection of the receiver operating characteristic (ROC) curve of a test accuracy study.

The H/B may be defined as the net harm (H) of treating a person without disease (i.e., false positive) to the net benefit (B) of treating a person with disease (i.e., true positive), the latter term equating to the net harm of a false negative result.^6^ The H/B ratio may be calculated from the Bayes’ equation as the product of the pretest odds of disease and the positive likelihood ratio at the specified test cutoff (which is equivalent to the slope of the ROC curve, TPR/FPR, at that point) and hence is equivalent to the post-test odds.^7^ A higher H/B ratio means the test is less likely to miss cases, and hence less likely to incur the harms of false negatives, and hence a higher H/B ratio is deemed better. Note that this scoring of H/B ratio may seem counterintuitive if one thinks solely of “harms” and “benefits,” hence the important qualification of “net”; to emphasize this point, henceforward it will be referred to as “net H/B ratio.”

More recently, another metric attempting to denote test limitation has been introduced: the LDM.^8,9^ LDM is based on “number needed” metrics which are generally deemed to be more intuitive and hence applicable for both clinicians and patients than Sens and Spec. One form of LDM is given by the ratio of the number needed to misdiagnose,^10^ which is the inverse of Inacc, to the number needed to diagnose, which is the inverse of Youden index. Hence, LDM may also be conceptualized as a ratio of harms (misdiagnosis) and benefits (diagnosis) and hence of the “fragility” of screening and diagnostic tests. LDM ranges from -1 to infinity but, as for likelihood ratios, has an inflection point at 1 such that LDM<1 indicates a test in which misdiagnosis is overall more likely than diagnosis and LDM>1 indicates a test in which diagnosis is overall more likely than misdiagnosis, and hence LDM>>1 is desirable and LDM=∞ is the perfect diagnostic test (where Sens=Spec=Y=1, and Inacc=0).

The purpose of this study was to compare these three indices of test limitation (MR, net H/B ratio, and LDM) for several brief CSIs in common clinical usage for dementia diagnosis, namely the Mini-Mental State Examination (MMSE),^11^ the Montreal Cognitive Assessment (MoCA),^12^ the Mini-Addenbrooke’s Cognitive Examination (MACE),^13^ the Six-item Cognitive Impairment Test (6CIT),^14^ the informant Ascertain Dementia 8 (iAD8),^15^ and the Test Your Memory test (TYM),^16^ as well as for a more recently described instrument, Free-Cog.^17^

## METHODS

### Participants

Data from previously undertaken and reported pragmatic prospective test accuracy studies in consecutive patient cohorts from a single clinic were reanalyzed (Table 2). In all studies, subjects had given informed consent and study protocol was approved by the institute’s committee on human research.

**Table 2.** Study demographics.

CSI	n	Age, median (years)	Gender (F:M; %F)	Pre-test odds of dementia
MMSE	244	60	117:127; 48	0.22
MoCA	260	59	118:142; 45	0.21
MACE	755	60	352:403; 47	0.18
6CIT	245	59	121:124; 49	0.25
AD8	212	64.5	106:106; 50	0.43
TYM	224	62	94:130; 42	0.54
Free-Cog	141	62	61:80; 43	0.12

CSI: cognitive screening instrument; MMSE: Mini-Mental State Examination; MoCA: Montreal Cognitive Assessment; MACE: Mini-Addenbrooke’s Cognitive Examination; 6CIT: Six-item Cognitive Impairment Test; AD8: Ascertain Dementia 8; TYM: Test Your Memory test.

### Procedures

The studies examined seven CSIs which were in routine use in a dedicated cognitive disorders clinic at different times: MMSE,^18,19^ MoCA,^20^ MACE,^21^ 6CIT,^22^ iAD8,^23^ TYM,^24^ and Free-Cog.^25^ Each of these base studies was undertaken using a standardized methodology in the cognitive disorders clinic which was located in a regional neuroscience center. Criterion diagnosis of dementia followed standard diagnostic criteria (DSM-IV) and was made independent of scores on CSIs to avoid review bias. Cross classification of criterion diagnosis with CSI test result, dichotomized by test cutoff, in a standard 2×2 contingency table allowed all cases to be classified as true positive (TP), false positive (FP), false negative (FN), and true negative (TN). Where possible, test cutoffs documented in the respective index studies^11,12,13,14,15,16,17^ for each instrument were used to avoid bias.

### Statistical analysis

All studies followed either the STAndards for the Reporting of Diagnostic accuracy studies (STARD)^26^ or the derived guidelines specific for dementia studies, STARDdem,^2^ dependent on the exact date at which each test accuracy study was undertaken. Standard summary measures of test discrimination were calculated, namely, sensitivity and specificity, and positive and negative likelihood ratios (LR+, LR−; classified according to Jaeschke et al.).^1^ In addition, summary measures of test limitation were calculated, namely, MR,^5^ net H/B ratio,^7^ and LDM.^8,9^

## RESULTS

Examining measures of test discrimination (Table 3), many were highly sensitive (MoCA, Free-Cog, MACE, and AD8) but had low specificity (MoCA, MACE, and AD8). Positive likelihood ratios were qualitatively either slight (MoCA, MACE, AD8, and TYM) or moderate (6CIT, Free-Cog, and MMSE), none achieving the large or very large classification.

**Table 3.** Comparing metrics of test discrimination and test limitation for CSIs for diagnosis of dementia versus no dementia.

CSI (Cutoff)	Measures of test discrimination			Measures of test limitation		
	Sens	Spec	LR+	MR	Net H/B ratio	LDM
MMSE (<26/30)	0.86	0.64	2.37=moderate	0.50	0.52	1.56
MoCA (<26/30)	1.00	0.31	1.46=slight	0.69	0.30	0.54
MACE (≤25/30)	0.99	0.32	1.45=slight	0.69	0.26	0.53
6CIT (>9/28)	0.88	0.78	4.00=moderate	0.34	1.00	3.30
AD8 (≥2/8)	0.98	0.10	1.09=slight	0.92	0.47	0.12
TYM (≤42/50)	0.95	0.45	1.73=slight	0.60	0.93	1.08
Free-Cog (≤22/30)	1.00	0.67	3.07=moderate	0.33	0.38	2.31

CSI: cognitive screening instrument; MMSE: Mini-Mental State Examination; MoCA: Montreal Cognitive Assessment; MACE: Mini-Addenbrooke’s Cognitive Examination; 6CIT: Six-item Cognitive Impairment Test; AD8: Ascertain Dementia 8; TYM: Test Your Memory test; Sens: sensitivity; Spec: specificity; LR+: positive likelihood ratio; MR: misclassification rate; H/B: net harm/net benefit ratio; LDM: likelihood to be diagnosed or misdiagnosed.

Examining measures of test limitation (Table 3), few achieved a MR of ≤0.5 (Free-Cog, 6CIT, and MMSE). Only one test (6CIT) achieved net H/B ratio of 1. LDM values of <1 (likelihood of misdiagnosis greater than correct diagnosis) were recorded for some tests (MoCA, MACE, and AD8). Of note, the tests with high sensitivity but low specificity generally fared worse on these metrics examining test limitation, while those with a better balance of Sens and Spec (reflected in the higher LR+s) did better. This was also evident in the overall ranking of CSIs by outcome of the examined measures of discrimination and limitation (Table 4).

**Table 4.** Ranking of cognitive screening instruments by outcome measures of test discrimination and test limitation (1=best, 7=worst).

Rank	Measures of test discrimination			Measures of test limitation		
	Sens	Spec	LR+	MR	Net H/B ratio	LDM
1	MoCA, Free-Cog	6CIT	6CIT	Free-Cog	6CIT	6CIT
2	–	Free-Cog	Free-Cog	6CIT	TYM	Free-Cog
3	MACE	MMSE	MMSE	MMSE	MMSE	MMSE
4	AD8	TYM	TYM	TYM	AD8	TYM
5	TYM	MACE	MoCA	MACE, MoCA	Free-Cog	MoCA
6	6CIT	MoCA	MACE	–	MoCA	MACE
7	MMSE	AD8	AD8	AD8	MACE	AD8

CSI: cognitive screening instrument; MMSE: Mini-Mental State Examination; MoCA: Montreal Cognitive Assessment; MACE: Mini-Addenbrooke’s Cognitive Examination; 6CIT: Six-item Cognitive Impairment Test; AD8: Ascertain Dementia 8; TYM: Test Your Memory test; Sens: sensitivity; Spec: specificity; LR+: positive likelihood ratio; MR: misclassification rate; H/B: net harm/net benefit ratio; LDM: likelihood to be diagnosed or misdiagnosed.

## DISCUSSION

The metrics examined here explicitly acknowledge test shortcomings, hence their designation as measures of test limitation in distinction from measures of test discrimination. Although limitation may be implicit in the latter (e.g., FNR in Sens, FPR in Spec), this inherent quality may not be apparent on a cursory examination. Moreover, some test metrics choose the best quality of a test and largely ignore its weaknesses (e.g., diagnostic odds ratio, area under the ROC curve) giving the most optimistic results. The measures of limitation examined here are seldom used in clinical practice, may be unfamiliar to clinicians, and have no exact ranges. Other methods of assessing test effectiveness and limitation are also available. The metrics examined here do not address utilities^7^ or cost ratios.^27^

This study has various shortcomings. The findings are of course dependent upon the diagnostic test accuracy studies upon which they are based.^18,19,20,21,22,23,24,25^ These base studies obviously have limitations, for example, they were undertaken in different patient populations, albeit all seen in the same cognitive disorders clinic and operating the same diagnostic criteria for dementia, and hence may not necessarily be generalizable. As the study setting was tertiary care, the data can only provide recommendations on optimal test for this setting and not necessarily for primary care where pretest odds of dementia would be lower. No information on patient education was collected in the base studies and hence test thresholds were not adjusted for educational level which may influence test performance.^28^ Nevertheless the findings suggest significant limitations for many of the CSIs in common usage. The findings might be corroborated by undertaking similar analyses with data reported in systematic reviews of these CSIs where available.

For MR and the net H/B ratio, lower or higher values, respectively, may be better, but precisely how high or how low is most desirable or optimal has not been defined. LDM values have clearer implications around the inflection point of 1. The influence of disease prevalence on MR is unknown, but as it is based (like Sens, Spec, FPR, and FNR) on strict columnar ratios from the 2×2 contingency table it is notionally uninfluenced by the base rate. Likewise, net H/B ratio is a function of LR+, which is also algebraically unrelated to the base rate. However, it is well recognized that these measures (Sens, Spec, and LR) are affected by the heterogeneity (spectrum bias) of clinical populations.^29^ Another formulation of LDM, with the denominator based on predictive values, takes account of disease prevalence.^8,9^

While clinicians may be content to use highly sensitive tests, accepting false positives as a reasonable tradeoff to ensure no cases are missed (i.e., low false negative rate), metrics of limitation highlight the potential shortcomings of such tests, and emphasize the need to find better tests. Patients undergoing testing may also want to have easily assimilated information on how well the test performs (a false positive diagnosis may have more significance for a patient than for a clinician) as well as its potential risks. Newer biomarker tests of dementia disorders could be subjected to similar analyses of test limitation.

In summary, CSIs have shortcomings which may be expressed using various metrics of limitation, as shown in this study. These complement the more familiar metrics of discrimination. Ideally, both should be examined by clinicians when deciding on optimal test selection according to setting and casemix.
